# Gut Microbiota-Mediated NLRP12 Expression Drives the Attenuation of Dextran Sulphate Sodium-Induced Ulcerative Colitis by Qingchang Wenzhong Decoction

**DOI:** 10.1155/2019/9839474

**Published:** 2019-04-02

**Authors:** Zhongmei Sun, Wenjing Pei, Yi Guo, Zhibin Wang, Rui Shi, Xiaowei Chen, Xingjie Zhao, Chen Chen, Jiali Liu, Xiang Tan, Wenjing Yuan, Tangyou Mao

**Affiliations:** ^1^Gastroenterology Department, Dongfang Hospital, Beijing University of Chinese Medicine, No. 6, 1st Section, Fangxingyuan, Fangzhuang, Beijing 100078, China; ^2^Beijing University of Chinese Medicine, No. 11, North Third Ring East Road, Beijing 100029, China; ^3^Gastroenterology Department, Dongzhimen Hospital, Beijing University of Chinese Medicine, No. 5, Haiyuncang Hutong, Beijing 100700, China

## Abstract

Qingchang Wenzhong Decoction (QCWZD) is a newly developed, effective traditional Chinese herbal formulation for ulcerative colitis (UC). In earlier studies, we found that QCWZD could relieve the clinical symptoms of UC patients, reduce inflammation, and improve the intestinal barrier function in dextran sulphate sodium (DSS)-induced UC rats. However, the relationship between QCWZD and the gut microbiota in colitis was not clarified. In this study, we established a rat model of DSS-induced UC and then investigated the regulatory effects of QCWZD on the gut microbiota using 16S rRNA analysis. We also determined the expression of NLRP12 after QCWZD administration. Our findings suggested that QCWZD administration could modulate gut microbiota composition and selectively promote the protective strains such as* Butyricimonas*,* Blautia,* and* Odoribacter, *whereas the enteric pathogens including* Clostridium* and* Dorea* were significantly reduced after QCWZD treatment. It is noteworthy that QCWZD administration was identified to promote gut microbiota-mediated NLRP12 expression by inhibiting the activity of the TLR4/Blimp-1 axis. In conclusion, our study supports the potential of QCWZD administration as a beneficial therapeutic strategy for UC.

## 1. Introduction

Ulcerative colitis (UC), a major clinical phenotype of inflammatory bowel disease, is an immunological disorder affecting mainly the rectum and colon. This disease seriously influences the quality of life of millions of patients throughout the world [1]. Currently, 5-amino salicylic acid, immunosuppressive drugs, corticosteroids, and biological agents form the primary therapeutic regimen for controlling inflammation in UC patients. However, besides having poor efficacy, these drugs are expensive and have considerable side effects such as intolerance or allergy responses [2]. Therefore, it is extremely important to explore new alternative strategies.

Traditional Chinese medicine (TCM) has the characteristics of flexible medication and less toxic and side effects, so it is widely used in the treatment of UC and especially plays a key role in regulating the gut microbiota. Huangqin decoction has been shown to ameliorate inflammation in the DSS-induced colitis through alteration of the gut microbiota, characterized by an increase in the relative abundance* Lactococcus* and a decrease in the numbers of* Desulfovibrio* and* Helicobacter *[3]. Traditional Herbal Medicine-Derived Sulforaphene LFS-01 demonstrated a significant effect on UC by selectively altering the gut microbiota and promoting intestinal gamma-delta T cells [4]. Qingchang Wenzhong Decoction (QCWZD) is a potent traditional Chinese herbal formulation formulated by Junxiang Li. Our previous studies found that QCWZD could relieve the clinical symptoms of UC patients [5, 6], reduce inflammation, and improve the intestinal barrier function in dextran sulphate sodium (DSS)-induced UC rats [7, 8]. Recently we found that there was a difference in the composition and abundance of gut microbiota in patients with UC and healthy people [9]; we wonder if QCWZD could treat UC by regulating gut microbiota, which is involved in the occurrence and development of UC [10].

Growing evidences indicate an association of gut microbiota with UC. Thousands of bacteria reside in the mammalian gastrointestinal tract, including more than 800 genera and 7,000 strains. These gut microbiota are mainly distributed in the intestinal cavity, mucosal surface, and intestine-related lymphoid tissue [11]. Gut microbiota comprise a major component of the gut microecosystem, which maintains a dynamic and stable physiological balance and plays an important role in maintaining intestinal physiological function, regulating immunity, and antagonizing pathogenic microorganism colonization [12, 13]. Increasing evidence shows that disrupted intestinal microbiota might be a contributing factor for UC by changing its quantity and diversity, participating in substance metabolism and regulating the intestinal mucosal immune inflammatory response [14, 15]. Therefore, restoring the intestinal microecological balance, suppressing intestinal inflammation, and restoring microbiome–host interactions could constitute a novel approach to treat UC [16].

NACHT, LRR, and PYD domains-containing protein 12 (NLRP12) is a member of the Nod-like receptor (NLR) family of proteins, which is a family of intracellular immune receptors that can activate caspase-1 by recognizing pathogens and damage-related molecules to form inflammatory bodies and participate in inflammatory responses to pathogen infection [17]. A recent study [18] demonstrated that NLRP12 was significantly downregulated in patients with active UC compared to its expression in healthy subjects and patients with inactive UC. Meanwhile, NLRP12-deficient mice are highly sensitive to dextran sulphate sodium (DSS)-induced colitis and exhibit exacerbated colonic inflammation [19, 20]. In parallel, gut dysbiosis caused by LPS-rich bacteria in the intestinal tract leads to intestinal leakage of the LPS endotoxin, thereby specifically stimulating the high expression of TLR4 [21]. TLR4 overexpression can induce the upregulation of B lymphocyte-induced maturation protein-1 (Blimp-1)—a zinc finger-containing transcriptional repressor that downregulates NLRP12 expression [22, 23]. Thus, reversing dysbiosis and promoting the expression of NLRP12 are important strategies for UC.

Therefore, we established a rat model of DSS-induced colitis to investigate the regulatory effects of QCWZD on the gut microbiota, as well as the expression of intestinal NLRP12 in these rats, in order to further clarify the regulation effect of QCWZD on inflammatory reaction of intestinal mucosa in UC.

## 2. Materials and Methods

### 2.1. Preparation of QCWZD

The constitution of QCWZD was as follows: 6 g of Huanglian (coptis), 10 g of PaoJiang (ginger), 15 g of Kushen (matrine), 6 g of Qingdai (indigo naturalis), 30 g of Diyutan (sanguisorba carbon), 6 g of Muxiang (wood), 6 g of Sanqi (pseudoginseng), and 6 g of Gancao (licorice). All herbal formulation granules were provided by the Pharmacy Department of Dongfang Hospital, Beijing University of Chinese Medicine (Beijing, China). 

### 2.2. Animal Model of Colitis and Treatment

All procedures involving animals were approved by the Animal Ethics Committee of Beijing University of Chinese Medicine. Male Sprague–Dawley rats (weight, 180–220 g) were purchased from the SPF Biotechnology Co., Ltd. (Beijing, China; certificate no. SCXK-2016-0002). The rats were adapted for 1 week prior to the experiments and were randomly divided into three groups (n = 8 per group): control group, DSS group, and DSS+QCWZD group. Specifically, a total of 24 rats were raised, of which 4 rats shared a cage. For the DSS group and DSS+QCWZD group, all 16 rats were given drinking water containing 4.5% (w/v) DSS (MP Biomedicals, MW: 36,000–50,000) ad libitum for 7 days to induce colitis. But in order to minimize the impact of cage effect on gut microbiota, half of the rats in a cage were treated with 0.3 g/kg-body weight QCWZD via oral gavage once a day, and the other half in the same cage were treated with same volume of distilled water. For the control group, the rats received the same volume of distilled water via oral gavage once a day, along with drinking water. Then all rats were sacrificed on day 8 for further analysis.

### 2.3. Fecal DNA Isolation and Library Construction

Fresh stool samples were collected on day 8, and microbial DNA was extracted using the E.Z.N.A.® soil DNA kit (Omega Bio-tek, Norcross, GA, USA) according to the manufacturer's protocols. The final DNA concentration was measured by a NanoDrop 2000 UV-vis spectrophotometer (Thermo Scientific, Wilmington, USA). The V3–V4 hypervariable regions of the bacterial 16S rRNA gene were amplified with the primers 338F (5′-ACTCCTACGGGAGGCAGCAG-3′) and 806R (5′-GGACTACHVGGGTWTCTAAT-3′) by a thermocycler (GeneAmp 9700; ABI, USA). PCR was conducted using the following program: 3 min of denaturation at 95°C; 27 cycles of 30 s at 95°C, 30 s for annealing at 55°C, and 45 s for elongation at 72°C; and a final extension at 72°C for 10 min. PCR was performed in triplicate in a 20 *μ*L mixture containing 4 *μ*L of 5X FastPfu Buffer, 2 *μ*L of 2.5 mM dNTPs, 0.8 *μ*L of the forward primer (5 *μ*M), 0.8 *μ*L of the reverse primer (5 *μ*M), 0.4 *μ*L of FastPfu polymerase, 0.2 *μ*L of BSA, and 10 ng of template DNA. The resultant PCR products were extracted from a 2% agarose gel and further purified using the AxyPrep DNA gel extraction kit (Axygen Biosciences, Union City, CA, USA) and quantified using QuantiFluor™-ST (Promega, USA) according to the manufacturer's protocol. DNA quality was checked by 1% agarose gel electrophoresis.

### 2.4. Bioinformatics Analysis

Purified amplicons were pooled in equimolar solutions and subjected to paired-end sequencing (2 × 300) on an Illumina MiSeq platform (Illumina, San Diego, CA, USA) according to the standard protocols. The analyses were conducted by following the “moving picture tutorial” and “Atacama soil microbiome tutorial” of Qiime2docs along with customized program scripts. Briefly, Raw FastQ files were demultiplexed and the quality examined by the demux plugin of Qiime2. The Amplicon Sequence Variant (ASV) table was generated by the DADA2 plugin of Qiime2, which performs paired-end read stitching, quality filtering, and chimeric variant filtering. The taxonomy of each representative sequence of ASV was annotated by the Sklearn classifier algorithm against Greengenes database version 13_8 (99% OTU dataset). Community diversity was measured by the Shannon–Weiner biodiversity index (Shannon index). Venn diagram analysis and principal coordinate analysis (PCoA) were then used to analyze the diversity between groups. LDA effect size (LEfSe) was used to detect dominant bacterial community differences among the three groups.

### 2.5. Western Blot Analysis

Western blot analysis was performed as described previously [24]. Total proteins from colonic tissues were extracted, and the protein concentrations were determined using the bicinchoninic acid assay (Cwbiotech, Beijing, China). Subsequently, the proteins were transferred to polyvinylidene fluoride membranes after being separated using 10% SDS-PAGE for 1.5 h. Next, the membranes were incubated overnight at 4°C with antibodies against NLRP12 (OM285742, 1:500), p-ERK (OM251946, 1:200), TLR4 (OM293436, 1:200), Blimp-1 (OM272531, 1:500), and *β*-actin (TA-09, 1:1000). All membranes were washed thrice for 10 min each, followed by incubation with secondary antibodies (Jackson, MS, USA) for 1 h at 37°C. Finally, densitometry analysis was performed by an imaging system (Gel Image System ver. 4.00, Tanon, China).

### 2.6. Real-Time PCR Analysis

Total RNA was extracted by using a Total RNA isolation kit (Cwbiotech). Quantitative real-time PCR (qPCR) was performed using the TRIzol method (TRIzol reagent; Invitrogen Life Technologies, Carlsbad, CA, USA) after reverse transcription into cDNA. The qPCR primer sequences for the target genes were as follows: 5′-GGGTCTGAATGACCTTGGGG-3′/5′-ACCTGGAGCACACTAGCAAC-3′ for NLRP12, 5′-AGAATCCAGGCTTCCTGCAC-3′/5′- AGGCTTGCTGGATGAGGTTC-3′ for MLCK, 5′-ACTGGGTGAGAAACGAGCTG-3′/5′-CAGCAATGGCTACACCAGGA-3′ for TLR4, 5′-GCTCTGGAAAGACCCTGACC-3′/5′-CCTTCTTGTGGAGCAGCAGA-3′ for Blimp-1, and 5′-CTTCCAGCCTTCCTTCCTGG-3′/5′-AATGCCTGGGTACATGGTGG-3′ for *β*-actin. Relative expression was assessed by using the comparative CT method (2^−ΔΔCt^).

### 2.7. Cytokine Measurements

After blood samples were collected from the abdominal aortas of rats, IL-1*β* and IL-18 levels in the serum were measured using ELISA kits (MULTISKAN MK3, Thermo, USA).

### 2.8. Statistical Analysis

All experimental results were expressed as the mean ± SEM. N refers to the number of mice used. GraphPad Prism 5 (Graph Pad Software, La Jolla, CA, USA) was used for statistical analyses. The data were compared between groups using one-way ANOVA, followed by Student's* t*-tests or Tukey's post hoc test. P < 0.05 was considered significant.

## 3. Results

### 3.1. QCWZD Shifted the Structure and Composition of Gut Microbiota in DSS-Induced UC Rats

**Figure 1:**
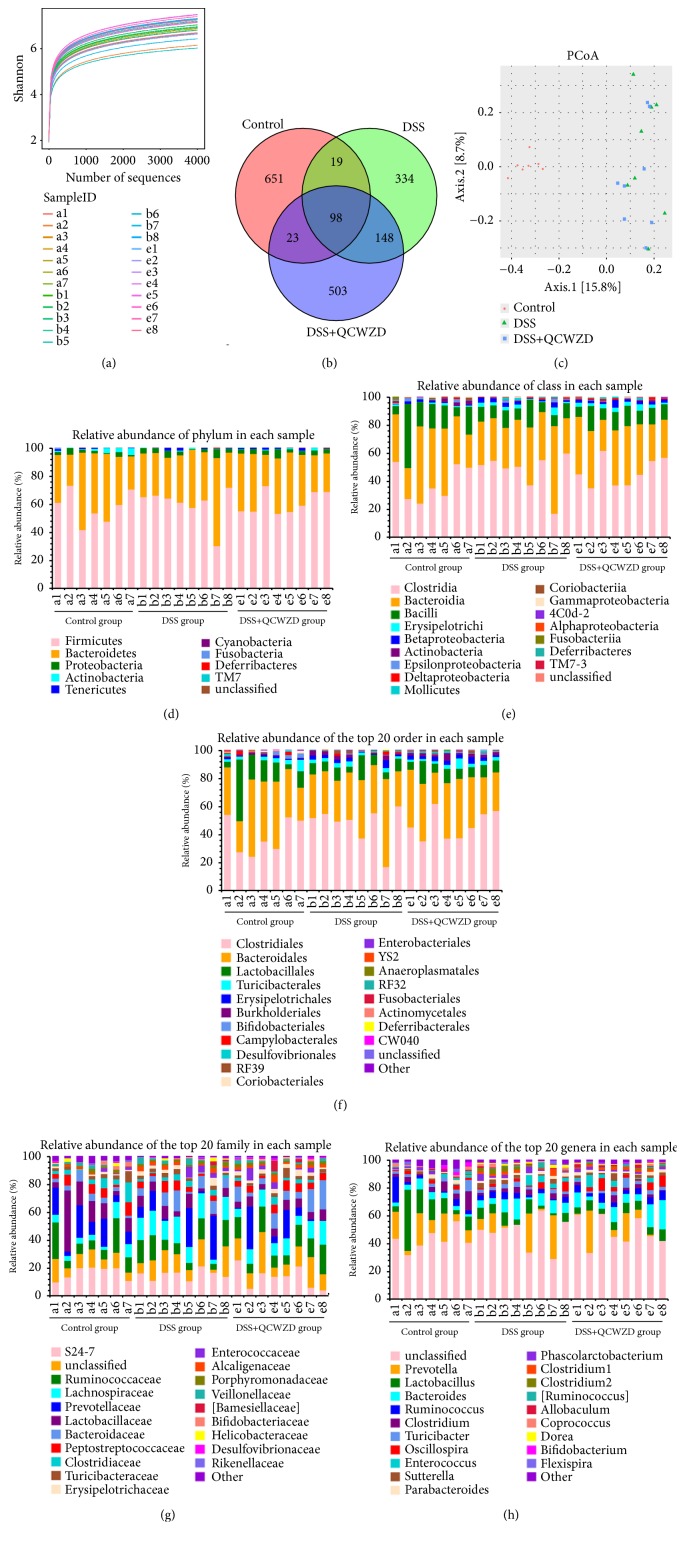
QCWZD treatment modulated gut microbiota structure in DSS-induced UC rats. (a) Shannon diversity curves of all samples. (b) Venn diagram of OTUs of each group. (c) Multiple sample PCoA analysis. (d) Relative abundance of phylum in each sample. (e) Relative abundance of class in each sample. (f) Relative abundance of the top 20 order in each sample. (g) Relative abundance of the top 20 family in each sample. (h) Relative abundance of the top 20 genera in each sample. Control, control group; DSS, DSS group; DSS+QCWZD, DSS+ Qingchang Wenzhong Decoction group.

Rat no. 8 in the control group died of unknown causes on day 3, the remaining rats tolerated the entire experiment well. To determine whether QCWZD has a regulatory effect on gut microbiota, we used bacterial 16S rRNA gene sequencing to analyze overall structural changes in the gut microbiota from DSS-induced UC rats after QCWZD treatment. In total, 927,822 usable reads and 1,776 OTUs were obtained from the 23 samples. As shown in Figure 1(a), Shannon diversity curves indicated that most of the diversity was captured in all samples. Then, we implemented a species Venn diagram analysis. As shown in Figure 1(b), 98 OTUs coexisted in the three groups; 117 OTUs were present in both the control and DSS groups, 246 OTUs in the DSS and DSS+QCWZD groups, and 121 OTUs in the control and DSS+QCWZD groups. PCoA analyses revealed that the gut microbiota in the DSS group and DSS+QCWZD group were separated from those of the control group, whereas the distance between the DSS+QCWZD and control groups was lesser than that between the DSS and control groups (Figure 1(c)).

Next, we investigated the microbial species and their relative abundance by histograms, which reflect the community structure of the gut microbiota. As shown in Figure 1(d), all samples contained* Firmicutes*,* Bacteroidetes*, and* Proteobacteria* at the phylum level. A marked decrease in* Actinobacteria* in DSS-treated rats compared to the control group was observed, and* Deferribacteres* was detected in the DSS group but not in the control or DSS+QCWZD groups. At the class level, all samples contained* Clostridia*,* Bacteroidia*, and* Bacilli* among a total of 16 classes,* Betaproteobacteria* was found at higher levels in the DSS and DSS+QCWZD group compared with that in the control group, and* Coriobacteriia *was detected in the control and DSS+QCWZD groups, but not in the DSS group (Figure 1(e)). Subsequently, sequencing data identified 21 orders of microbial flora.* Burkholderiales *were identified at significantly higher levels in the DSS group compared to the control group (Figure 1(f)).* Coriobacteriales* and* Anaeroplasmatales* were not detected in the DSS group but were detected in the control and DSS+QCWZD group. Conversely,* RF32* was identified in the DSS group but not in the other groups. As shown in Figure 1(g),* S24-7*,* Ruminococcaceae*,* Lactobacillaceae*, and* Prevotellaceae* strains accounted for the majority of the 37 families of microbiota. Finally, 51 genera were identified in all samples. A marked increase in* Dorea* and* Sutterella* was detected in DSS-treated rats compared to the control group rats (Figure 1(h)).

### 3.2. QCWZD Regulated the Proliferation of Certain Bacteria in DSS-Induced UC Rats

**Figure 2:**
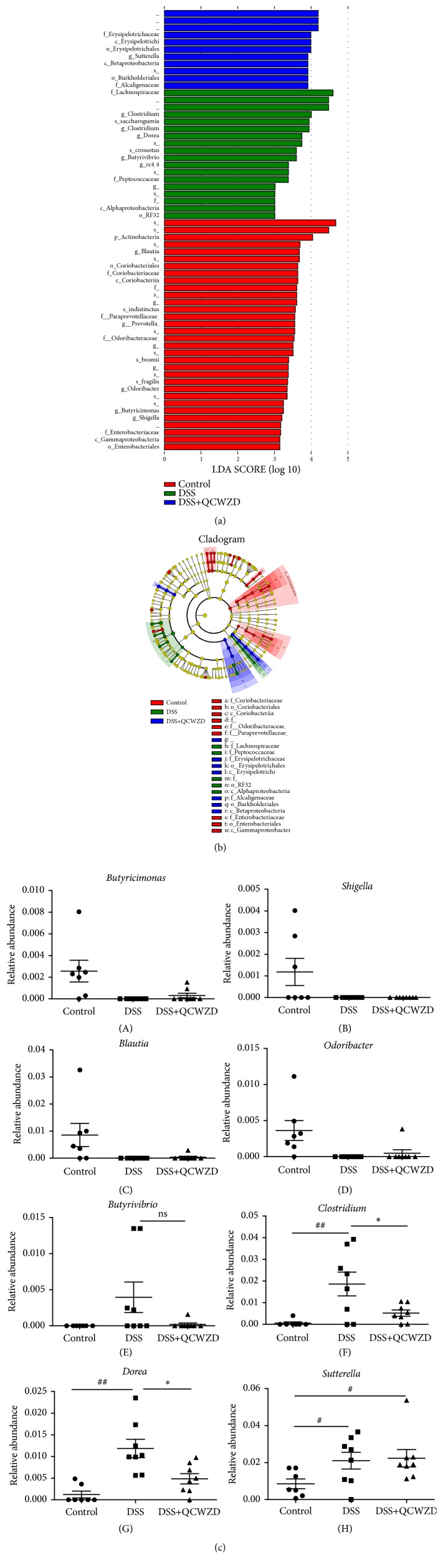
QCWZD regulated the proliferation of certain bacteria in DSS-induced UC rats. (a) Distribution histogram based on LDA. (b) Cladogram. (c) The relative abundances of bacterial groups at the genus level between groups. Control, control group; DSS, DSS group; DSS+QCWZD, DSS+ Qingchang Wenzhong Decoction group. ^##^ P < 0.01, ^#^P < 0.05 versus the control group; *∗* P < 0.05 versus the DSS group.

We identified biomarkers and dominant microbiota in the three groups by LEfSe. As shown Figure 2(a), the distribution histogram showed thirty-two taxa in the control group but not in the DSS and DSS+QCWZD groups.* Actinobacteria* was the predominant intestinal phylum. Eighteen taxa were found in the DSS group, and* Lachnospiraceae* constituted the predominant community members. Eleven taxa were found in the DSS+QCWZD group, and* Erysipelotrichaceae*,* Erysipelotrichi*, and* Erysipelotrichales* were the major microbiota. Then, we generated an evolutionary clustering analysis diagram based on the LDA score to identify important microbiota. As shown in Figure 2(b), the resulting cladogram revealed that the branches of* Actinobacteria*,* Paraprevotellaceae*,* Odoribacteraceae* and* Enterobacteriaceae*, and* Actinobacteria* represented the major microbiota in the control group. In the DSS group,* Peptococcaceae* and* Lachnospiraceae* played an important role in the development of UC. Interestingly,* Alcaligenaceae* were identified as the predominant microbiota in the DSS+QCWZD group (Figure 2(b)).

In addition, the results in Figure 2(c) showed* Butyrivibrio* was not detected in the control group but was detected in the DSS and DSS+QCWZD groups. The relative abundances of* Clostridium*,* Dorea,* and* Sutterella* were higher in the DSS group compared to the control group, whereas the relative abundances of* Clostridium* and* Dorea* were remarkably lower in the DSS+QCWZD group compared with the DSS group (Figure 2(c)). Interestingly,* Butyricimonas*,* Shigella*,* Blautia*, and* Odoribacter* were not detected in the DSS group, but* Butyricimonas*,* Blautia*, and* Odoribacter* were detected in the control and DSS+QCWZD group (Figure 2(c)). In summary, QCWZD administration could modulate gut microbiota composition and regulate the proliferation of certain bacteria, characterized by an increase in* Butyricimonas*,* Blautia*, and* Odoribacter* and decrease in* Clostridium* and* Dorea* in DSS-induced UC rats.

### 3.3. QCWZD Upregulated the NLRP12 Expression to Improve Intestinal Barrier and Suppress Intestinal Inflammation in DSS-Induced UC Rats

**Figure 3:**
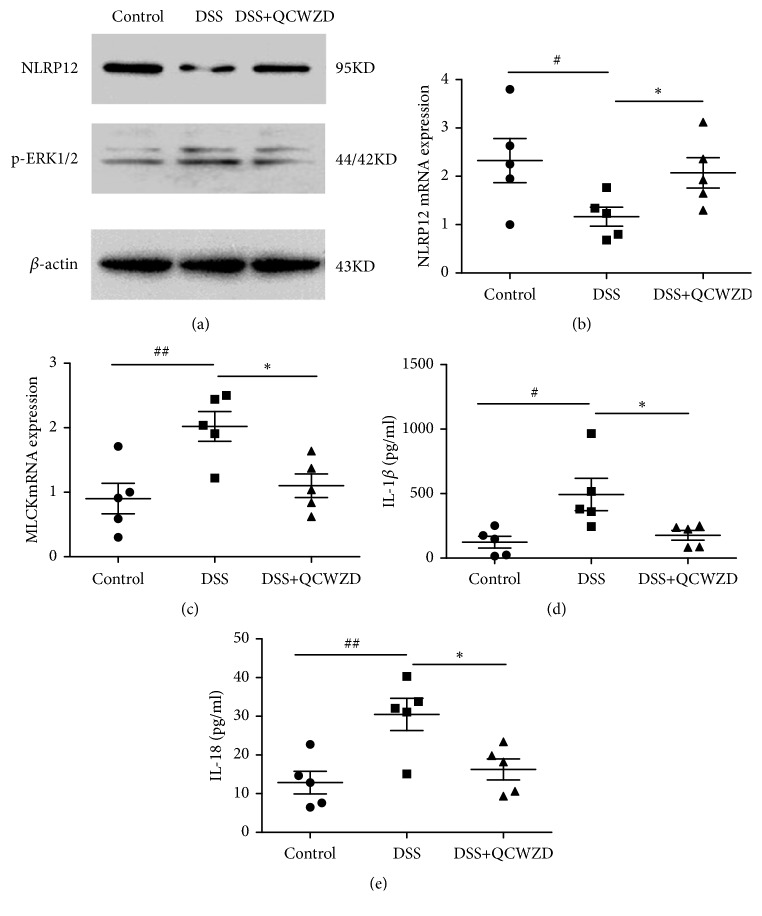
QCWZD upregulated the NLRP12 expression to improve intestinal barrier and suppress intestinal inflammation in DSS-induced UC rats. NLRP12 and p-ERK protein expression levels in colon tissues were analyzed by western blot analysis (a). NLRP12 (b) and MLCK (c) gene expression levels in colon tissues were analyzed by qPCR. IL-1*β* (d) and IL-18 (e) levels were detected by ELISA. Control, control group; DSS, DSS group; DSS+QCWZD, DSS+Qingchang Wenzhong Decoction group. ^##^ P < 0.01, ^#^P < 0.05 versus the control group; *∗∗* P < 0.01, *∗* P < 0.05 versus the DSS group.

A recent study determined a key role for NLRP12 in dictating intestinal inflammation and preventing colitis [25]; therefore, we examined the expression of NLRP12 by western blot and qPCR analyses. As shown in Figures 3(a) and 3(b), the NLRP12 level was significantly lower in the DSS group than in the control group (P < 0.05), and QCWZD treatment induced NLRP12 expression (P < 0.05, P < 0.01, respectively).

Extracellular signal-regulated kinases (ERK), a downstream gene of NLRP12 [26], regulates MLCK phosphorylation, which can increase the permeability of the intestinal mucosa and aggravate intestinal inflammation by regulating the expression of claudin, occludin, and ZO proteins [27]. Therefore, we analyzed p-ERK and MLCK levels. Western blot analyses showed that the p-ERK level was significantly higher in the DSS group than in the control group (P < 0.01, Figure 3(a)). A significantly lower p-ERK level was present in the DSS+QCWZD group than in the DSS group (P < 0.05). Similarly, qPCR analyses showed significantly higher MLCK mRNA levels in the DSS group than in the control group (P < 0.01, Figure 3(c)), and DSS+QCWZD group rats showed significantly lower levels of MLCK mRNA compared with the DSS group rats (P < 0.05).

NLRP12 was demonstrated to interfere with the release of proinflammatory cytokines, which participated in intestinal inflammatory reaction and promoted the development of colitis [18, 19]. Hence, we examined the expression levels of IL-1*β* and IL-18. As shown Figures 3(d) and 3(e), colonic IL-1*β* and IL-18 levels were significantly higher in the DSS group than in the control group (P<0.05, P<0.01, respectively). Compared with the DSS group, QCWZD administration significantly decreased levels of IL-1*β* and IL-18 (P < 0.05).

### 3.4. QCWZD Promoted Gut Microbiota-Mediated NLRP12 Expression by Inhibiting the TLR4/Blimp-1 Axis in DSS-Induced UC Rats

**Figure 4:**
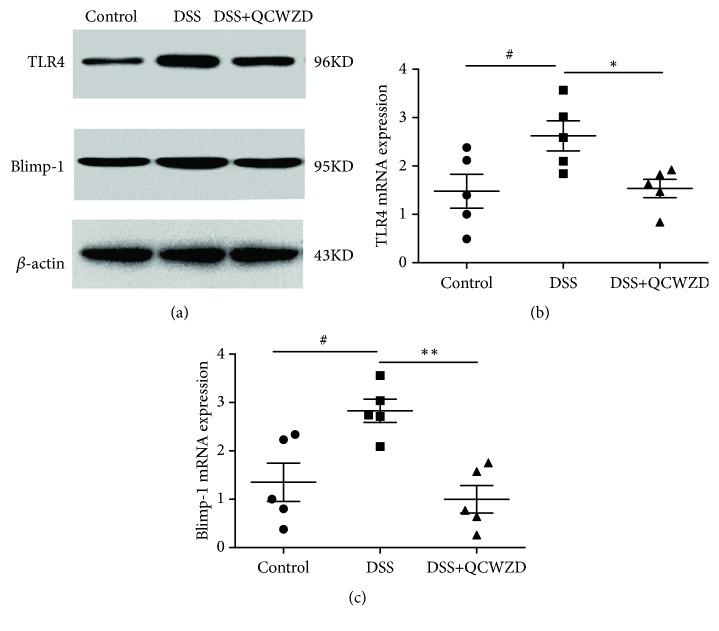
QCWZD promoted gut microbiota-mediated NLRP12 expression by inhibiting the TLR4/Blimp-1 axis in DSS-induced UC rats. TLR4 and Blimp-1 protein expression levels in colon tissues were analyzed by western blot analysis (a). TLR4 (b) and Blimp-1 (c) gene expression levels in colon tissues were analyzed by qPCR. Control, control group; DSS, DSS group; DSS+QCWZD, DSS+ Qingchang Wenzhong Decoction group. ^##^ P < 0.01, ^#^P < 0.05 versus the control group; *∗∗* P < 0.01, *∗* P < 0.05 versus the DSS group.

Gut dysbiosis can stimulate the expression of TLR4 and induce the upregulation of Blimp-1, thereby inhibiting the expression of NLRP12, which plays an important role in the development of UC [21, 22]. Therefore, we examined the expression of the TLR4/Blimp-1 axis. As shown in Figure 4(a), the induction of colitis by DSS significantly elevated colonic TLR4 and Blimp-1 gene and protein expression (P < 0.05, P < 0.01, respectively) compared with the control group. Treatment with QCWZD for 7 days decreased TLR4 and Blimp-1 gene expression (Figures 4(b) and 4(c), P < 0.05, P < 0.01, respectively).

## 4. Discussion

The dysbiosis of gut microbiota contributes to the pathogenesis of UC by causing intestinal inflammatory responses and damaging the intestinal mucosal barrier [12, 28]. Our previous studies found that QCWZD could relieve the clinical symptoms of UC patients [5, 6]; however, the specific mechanism of action was not clear. Because QCWZD is a compound of multiflavor Chinese medicines and each of them has different chemical constituents, it may have regulatory effects on UC through multilinks and multiple targets, so we carried out a number of studies. We found QCWZD can alleviate inflammatory reaction and improve the intestinal barrier function in DSS-induced UC rats by downregulating the IP10/CXCR3 axis [8] and upregulating the MSP/RON pathway [7]. Furthermore, we also found that QCWZD could resist the interactive network of inflammation, oxidative stress, apoptosis, and their overactivated interactions [29]. Recently we found that there was a significant difference in the composition and abundance of gut microbiota between UC patients and healthy people [9]. Among them,* Bacteroidaceae*,* Bacteroides, Firmicutes, *and* Clostridia* were the dominant groups in healthy people and* Lactobacillus*,* Lactobacillaceae*,* Erysipelotrichaceae,* and* Erysipelotrichales* were the major microbiota in UC patients. But the relationship between QCWZD and the gut microbiota in UC has not been elucidated. Therefore, we designed this study to investigate whether QCWZD ameliorates UC by regulating intestinal microecology.

The microbiota composition in DSS-induced UC rats was investigated by high-throughput sequencing. As shown in Figure 1(a), Shannon curves revealed that most of the diversity was captured in all samples, and the microbial diversity in the DSS and DSS+QCWZD groups was greater than that in the control group; however, the difference was not statistically significant. PCoA analyses revealed significant distances among the three groups, whereas the distance between the QCWZD and control groups was the least (Figure 1(c)). Subsequently, we detected the overall structure of gut microbiota in three groups on the phylum, class, order, family, and genus level.

Our results revealed that* Firmicutes*,* Bacteroidetes*, and* Proteobacteria* were the dominant phyla in the three groups, similar to previous studies [30]. A marked decrease in* Actinobacteria* in DSS-treated rats was found compared to the control group, but the DSS and DSS+QCWZD groups did not differ. The result that* Deferribacteres* occurred in the DSS group but not in the control or DSS+QCWZD groups suggested that QCWZD might be inhibiting the growth of members of this phylum in the gut. At the class level, the DSS group had a higher abundance of* Betaproteobacteria *and* Coriobacteriia *was not detected. Nevertheless, the proportions of* Coriobacteriia* were restored by QCWZD administration (Figure 1(e)). At the order level, we observed that the DSS group had a significantly increased abundance of* Burkholderiales*, and* RF32* was identified in the DSS group but not in the other groups;* Coriobacteriales* and* Anaeroplasmatales* were in the control and DSS+QCWZD group but not in the DSS groups. These results indicate that QCWZD administration recovered these skewed bacterial groups. Our findings also indicate that QCWZD can selectively promote the growth of protective strains, such as those of* Butyricimonas*,* Blautia*, and* Odoribacter*, consistent with studies that reported lower relative abundances of* Butyricimonas* and* Blautia* in the UC of patients with Crohn's disease than in healthy individuals without inflammatory bowel disease [31–33].* Butyricimonas*, a negative correlation with proinflammatory cytokines [34], was lower in both the noninflamed and inflamed sites of UC patients compared to the corresponding site of non-IBD controls, suggesting that it may play a key protective role in the occurrence and development of UC [31].* Blautia is *one of the dominant butyrate-producing bacteria and plays a beneficial role in maintaining human health. Numerous studies have reported that a lower relative abundance of* Blautia *was found in UC individuals [31, 32, 35], which is inconsistent with our present study.* Odoribacter is also *a butyrate-producing bacteria and was found significantly lower in UC patients [34], whereas* Odoribacter* was of increased abundance in azoxymethane (AOM) and DSS-induced colitis associated cancer (CAC) in mice [36]. The reason behind the difference is that our result is based on DSS-induced UC model, while the study focused on AOM/DSS-induced CAC. Therefore, the relationship between* Odoribacter* and colitis needs to be investigated further. Moreover, we also observed a significant decrease in harmful enteric pathogens such as* Clostridium* and* Dorea* after QCWZD treatment, which is in accordance with previous studies [37–39]. Among them, numerous studies have shown that* Clostridium* difficile, a major strain of* Clostridium genus, *is a major cause of spontaneous colitis and antimicrobial agents such that fidaxomicin can relieve colitis [40, 41]. A previous study [42] found that the relative abundance of* Dorea* was significantly increased in the DSS-treated mice, which is consistent with our research, suggesting that it may promote the development of UC. The above results show that QCWZD administration could modulate gut microbiota composition and selectively promote the protective strains and suppress the enteric pathogens.

Recent studies have implicated that gut dysbiosis can stimulate the expression of TLR4 and induce the upregulation of Blimp-1, thereby inhibiting the expression of NLRP12, which is a negative immune regulator and plays a key role in modulating the diversity of the gut microbiome and maintaining intestinal homeostasis [21, 22, 43]; therefore, we also investigated whether QCWZD could influence the expression of NLRP12 and its downstream factors. Our results demonstrated that the induction of colitis significantly decreased colonic NLRP12 expression, which is consistent with studies that demonstrated the involvement of NLRP12 in the occurrence and development of UC [18, 22]. QCWZD treatment was found to induce NLRP12 expression (P < 0.05, P < 0.01, respectively). At the same time, QCWZD administration significantly decreased the levels of p-ERK, MLCK, IL-1*β*, and IL-18. Furthermore, increased expression of TLR4 during the imbalance of the gut microbiota has been demonstrated to promote the upregulation of Blimp-1, which has a negative regulatory effect on the expression of NLRP12 [21, 22]. Therefore, we examined the expression of TLR4/Blimp-1 axis. We found that QCWZD treatment decreased TLR4 and Blimp-1 gene expression (P < 0.05, P < 0.01, respectively). Collectively, the above results suggested that QCWZD ameliorates colonic inflammation by regulating NLRP12 expression during UC rat colitis via inhibiting TLR4/Blimp-1 axis action in response to altered microbiota composition.

In conclusion, QCWZD can protect against DSS-induced colitis by modulating gut microbiota and promoting NLRP12 expression, which occurs via the inhibition of the activity of the TLR4/Blimp-1 axis. However, due to the limitation of 16S rRNA analysis, we only discussed the regulation of QCWZD on the structure and composition of gut microbiota, but it is not clear whether the function and metabolism of microbiota could be regulated, and further study is needed.

## Data Availability

The data used to support the findings of this study are available from the corresponding author upon request.

## References

[1] Xu J., Chen N., Wu Z. (2018). 5-Aminosalicylic acid alters the gut bacterial microbiota in patients with ulcerative colitis. *Frontiers in Microbiology*.

[2] Zou Y., Li W. Y., Wan Z. (2015). Huangqin-Tang ameliorates TNBS-induced colitis by regulating effector and regulatory CD4^+^ T cells. *BioMed Research International*.

[3] Yang Y., Chen G., Yang Q. (2017). Gut microbiota drives the attenuation of dextran sulphate sodium-induced colitis by Huangqin decoction. *Oncotarget *.

[4] Li M., Gao J., Tang Y. (2017). Traditional herbal medicine-derived sulforaphene LFS-01 reverses colitis in mice by selectively altering the gut microbiota and promoting intestinal gamma-delta t cells. *Frontiers in Pharmacology*.

[5] Mao T., Chen J., Wei S. (2016). The treatment of 84 cases of ulcerative colitis with Qingchang Wenzhong Decoction. *Global Traditional Chinese Medicine*.

[6] Wang Z., Chen Ch., Guo Y. (2018). Therapeutic analysis of Qingchang Wenzhong Formula for treatment of mild-moderate ulcerative colitis patients. *Chinese Journal of Integrated Traditional and Western Medicine*.

[7] Mao T., Li J., Liu L. (2017). Qingchang Wenzhong Decoction attenuates DSS-induced colitis in rats by reducing inflammation and improving intestinal barrier function via upregulating the MSP/RON signalling pathway. *Evidence-Based Complementary and Alternative Medicine*.

[8] Mao T., Shi R., Zhao W. (2016). Qingchang Wenzhong Decoction ameliorates dextran sulphate sodium-induced ulcerative colitis in rats by downregulating the IP10/CXCR3 axis-mediated inflammatory response. *Evidence-Based Complementary and Alternative Medicine*.

[9] P. Ding (2018). Study on diversity of intestinal flora of patients with large intestine damp heat syndrome and ulcerative colitis by high-throughput sequencing. *Modernization of Traditional Chinese Medicine and Materia Medica-World Science and Technology*.

[10] Machiels K., Joossens M., Sabino J. (2014). A decrease of the butyrate-producing species *Roseburia hominis* and *Faecalibacterium prausnitzii* defines dysbiosis in patients with ulcerative colitis. *Gut*.

[11] Cresci G. A., Bawden E. (2015). Gut Microbiome. *Nutrition in Clinical Practice*.

[12] Bernstein C. N., Forbes J. D. (2017). Gut Microbiome in Inflammatory Bowel Disease and Other Chronic Immune-Mediated Inflammatory Diseases. *Inflammatory Intestinal Diseases*.

[13] Ni J., Wu G. D., Albenberg L., Tomov V. T. (2017). Gut microbiota and IBD: causation or correlation?. *Nature Reviews Gastroenterology & Hepatology*.

[14] Nishida A., Inoue R., Inatomi O., Bamba S., Naito Y., Andoh A. (2018). Gut microbiota in the pathogenesis of inflammatory bowel disease. *Journal of Clinical Gastroenterology*.

[15] Sun M., Wu W., Chen L. (2018). Microbiota-derived short-chain fatty acids promote Th1 cell IL-10 production to maintain intestinal homeostasis. *Nature Communications*.

[16] Smolinska S., O'Mahony L. (2016). Microbiome–Host Immune System Interactions. *Seminars in Liver Disease*.

[17] Rubino S. J., Selvanantham T., Girardin S. E., Philpott D. J. (2012). Nod-like receptors in the control of intestinal inflammation. *Current Opinion in Immunology*.

[18] Chen L., Wilson J. E., Koenigsknecht M. J. (2017). NLRP12 attenuates colon inflammation by maintaining colonic microbial diversity and promoting protective commensal bacterial growth. *Nature Immunology*.

[19] Allen I. C., Wilson J. E., Schneider M. (2012). NLRP12 suppresses colon infammation and tumorigenesis through the negative regulation of noncanonical NF-*κ*B signaling. *Immunity*.

[20] Zaki M. H., Vogel P., Malireddi R. K. S. (2011). The NOD-like receptor NLRP12 attenuates colon inflammation and tumorigenesis. *Cancer Cell*.

[21] Fang J., Sun X., Xue B., Fang N., Zhou M. (2017). Dahuang Zexie decoction protects against high-fat diet-induced NAFLD by modulating gut microbiota-mediated Toll-like receptor 4 signaling activation and loss of intestinal barrier. *Evidence-Based Complementary and Alternative Medicine*.

[22] Zhu G., Wang H., Wang T., Shi F. (2017). Ginsenoside Rg1 attenuates the inflammatory response in DSS-induced mice colitis. *International Immunopharmacology*.

[23] Shi F., Yang Y., Kouadir M., Xu W., Hu S., Wang T. (2016). Inflammasome-independent role of NLRP12 in suppressing colonic inflammation regulated by Blimp-1. *Oncotarget*.

[24] Huang F., Kao C.-Y., Wachi S., Thai P., Ryu J., Wu R. (2007). Requirement for both JAK-mediated PI3K signaling and ACT1/TRAF6/TAK1- dependent NF-*κ*B activation by IL-17A in enhancing cytokine expression in human airway epithelial cells. *The Journal of Immunology*.

[25] Ray K. (2017). Gut microbiota: NLRP12 regulates gut microbiota to suppress intestinal inflammation. *Nature Reviews Gastroenterology & Hepatology*.

[26] Wang Q., Liu F., Zhang M. (2018). NLRP12 promotes mouse neutrophil differentiation through regulation of non-canonical NF-*κ*B and MAPK ERK1/2 signaling. *International Journal of Biological Sciences*.

[27] Al-Sadi R., Guo S., Ye D., Ma T. Y. (2013). TNF-*α* modulation of intestinal epithelial tight junction barrier is regulated by ERK1/2 activation of Elk-1. *The American Journal of Pathology*.

[28] Shen Z.-H., Zhu C.-X., Quan Y.-S. (2018). Relationship between intestinal microbiota and ulcerative colitis: mechanisms and clinical application of probiotics and fecal microbiota transplantation. *World Journal of Gastroenterology*.

[29] Shi L., Dai Y., Jia B. (2018). The inhibitory effects of Qingchang Wenzhong granule on the interactive network of inflammation, oxidative stress, and apoptosis in rats with dextran sulfate sodium‐induced colitis. *Journal of Cellular Biochemistry*.

[30] Cui H., Cai Y., Wang L. (2018). Berberine regulates Treg/Th17 balance to treat ulcerative colitis through modulating the gut microbiota in the colon. *Frontiers in Pharmacology*.

[31] Hirano A., Umeno J., Okamoto Y. (2018). Comparison of the microbial community structure between inflamed and non-inflamed sites in patients with ulcerative colitis. *Journal of Gastroenterology and Hepatology*.

[32] Chen G., Zhang Y., Wang W. (2017). Partners of patients with ulcerative colitis exhibit a biologically relevant dysbiosis in fecal microbial metacommunities. *World Journal of Gastroenterology*.

[33] Wang Y., Gao X., Ghozlane A. (2018). Characteristics of faecal microbiota in paediatric crohn’s disease and their dynamic changes during infliximab therapy. *Journal of Crohn's and Colitis*.

[34] Jangi S., Gandhi R., Cox L. M. (2016). Alterations of the human gut microbiome in multiple sclerosis. *Nature Communications*.

[35] Nishino K., Nishida A., Inoue R. (2018). Analysis of endoscopic brush samples identified mucosa-associated dysbiosis in inflammatory bowel disease. *Journal of Gastroenterology*.

[36] Song H., Wang W., Shen B. (2018). Pretreatment with probiotic Bifico ameliorates colitis-associated cancer in mice: Transcriptome and gut flora profiling. *Cancer Science*.

[37] Ohkawara S., Furuya H., Nagashima K., Asanuma N., Hino T. (2006). Effect of oral administration of butyrivibrio fibrisolvens MDT-1 on experimental enterocolitis in mice. *Clinical and Vaccine Immunology*.

[38] Micic D., Hirsch A., Setia N., Rubin D. T. (2018). Enteric infections complicating ulcerative colitis. *Intestinal Research*.

[39] Lee T., Clavel T., Smirnov K. (2015). Oral versus intravenous iron replacement therapy distinctly alters the gut microbiota and metabolome in patients with IBD. *Gut*.

[40] Higa J. T., Kelly C. P. (2014). New drugs and strategies for management of *Clostridium difficile* colitis. *Journal of Intensive Care Medicine*.

[41] Kang Y., Yang G., Zhang S. (2018). Goji berry modulates gut microbiota and alleviates colitis in il-10-deficient mice. *Molecular Nutrition & Food Research*.

[42] Praengam K., Sahasakul Y., Kupradinun P. (2017). Brown rice and retrograded brown rice alleviate inflammatory response in dextran sulfate sodium (DSS)-induced colitis mice. *Food & Function*.

[43] Lau J. M.-F., Dombrowski Y. (2018). The innate immune receptor NLRP12 maintains intestinal homeostasis by regulating microbiome diversity. *Cellular & Molecular Immunology*.

